# Challenges in applying the GRADE approach in public health guidelines and systematic reviews: a concept article from the GRADE Public Health Group

**DOI:** 10.1016/j.jclinepi.2021.01.001

**Published:** 2021-07

**Authors:** Michele Hilton Boon, Hilary Thomson, Beth Shaw, Elie A. Akl, Stefan K. Lhachimi, Jesús López-Alcalde, Miloslav Klugar, Leslie Choi, Zuleika Saz-Parkinson, Reem A. Mustafa, Miranda W. Langendam, Olivia Crane, Rebecca L. Morgan, Eva Rehfuess, Bradley C. Johnston, Lee Yee Chong, Gordon H. Guyatt, Holger J. Schünemann, Srinivasa Vittal Katikireddi, GRADE Working Group

**Affiliations:** a MRC/CSO Social and Public Health Sciences Unit, Berkeley Square, 99 Berkeley Street, University of Glasgow, Glasgow G3 7HR, UK; b Center for Evidence-based Policy, Oregon Health & Science University, Portland, OR 97201 USA; c Department of Health Research Methods, Evidence, and Impact, McMaster University, 1280 Main Street W, Hamilton, Ontario L8S 4K1, Canada; d Department of Internal Medicine, American University of Beirut, Beirut, Lebanon; e Department for Health Services Research, Institute of Public Health and Nursing Research, University of Bremen, Grazer Straße 4, 28359 Bremen, Germany; f Health Sciences Bremen, University of Bremen, 28359 Bremen, Germany; g Department of Paediatrics, Obstetrics & Gynaecology and Preventative Medicine, Universitat Autònoma de Barcelona; Faculty of Health Sciences, Universidad Francisco de Vitoria (UFV)-Madrid; Clinical Biostatistics Unit, Hospital Universitario Ramón y Cajal (IRYCIS); CIBER Epidemiology and Public Health; Cochrane Associate Centre of Madrid, Madrid, Spain; h Faculty of Medicine, Czech National Centre for Evidence-Based Healthcare and Knowledge Translation (Cochrane Czech Republic, The Czech Republic Centre for Evidence-Based Healthcare; JBI Centre of Excellence, Masaryk University GRADE Centre), Institute of Biostatistics and Analyses, Masaryk University, 625 00 Brno, Czechia; i The Department of Vector Biology, Partnership for Increasing the Impact of Vector Control, Liverpool School of Tropical Medicine, Liverpool, UK; j European Commission, Joint Research Centre, Ispra, Italy; k Departments of Medicine and Biomedical & Health Informatics, University of Missouri-Kansas City, Kansas City, MO 66160 USA; l Department of Clinical Epidemiology, Amsterdam University Medical Centres, University of Amsterdam, Biostatistics and Bioinformatics, Amsterdam Public Health Research Institute, Amsterdam, the Netherlands; m National Institute for Health and Care Excellence (NICE), Level 1A, City Tower, Piccadilly Plaza, Manchester M1 4BT, UK; n Institute for Medical Informatics, Biometry and Epidemiology, Pettenkofer School of Public Health, LMU Munich, Munich, Germany; o Department of Nutrition, Texas A&M University, College Station, TX, USA; p Cochrane Public Health and Health Systems Network, University of Oxford, Oxford, UK; q Department of Health Research Methods, Michael G DeGroote Cochrane Canada and McMaster GRADE Centres, and WHO Collaborating Centre for Infectious Diseases, Research Methods and Recommendations, Evidence, and Impact, McMaster University, Hamilton, Ontario, Canada

**Keywords:** GRADE, Public health, Guidelines, Nonrandomized studies, Health policy, Social determinants

## Abstract

**Background and Objective:**

This article explores the need for conceptual advances and practical guidance in the application of the GRADE approach within public health contexts.

**Methods:**

We convened an expert workshop and conducted a scoping review to identify challenges experienced by GRADE users in public health contexts. We developed this concept article through thematic analysis and an iterative process of consultation and discussion conducted with members electronically and at three GRADE Working Group meetings.

**Results:**

Five priority issues can pose challenges for public health guideline developers and systematic reviewers when applying GRADE: (1) incorporating the perspectives of diverse stakeholders; (2) selecting and prioritizing health and “nonhealth” outcomes; (3) interpreting outcomes and identifying a threshold for decision-making; (4) assessing certainty of evidence from diverse sources, including nonrandomized studies; and (5) addressing implications for decision makers, including concerns about conditional recommendations. We illustrate these challenges with examples from public health guidelines and systematic reviews, identifying gaps where conceptual advances may facilitate the consistent application or further development of the methodology and provide solutions.

**Conclusion:**

The GRADE Public Health Group will respond to these challenges with solutions that are coherent with existing guidance and can be consistently implemented across public health decision-making contexts.

**What is new?**

**Key findings**
• Nine studies of guideline developers’ experiences reported challenges when using GRADE in public health and related fields.
• The GRADE Public Health project group will respond to these challenges by producing guidance relating to social determinants, population-level outcomes, and rating certainty of evidence from interrupted time series.

**What this adds to what was known**
• Thematic analysis indicates the challenges relate to diverse perspectives and outcomes, decision-making thresholds, and nonrandomized studies.
• Case studies drawing on Cochrane reviews and public health guidelines provide examples of how these challenges are encountered in practice.

**What is the implication and what should change now?**
• Detailed examples, new guidance, and targeted training materials should be developed to support public health and public policy audiences using GRADE.

## 1 Introduction

### 1.1 Overview

For the past decade, the GRADE Working Group has produced tools and guidance with the aim of reducing unnecessary confusion and variation in rating the certainty in synthesized evidence and improving the transparency and methodological rigor of guidelines. Several GRADE outputs and project groups have specifically addressed public health topics and used examples from public health guidelines [[1], [2], [3]]. At the same time, public health guideline developers and others using GRADE in public health-related contexts have identified ongoing challenges. To address these challenges and consider the solutions, the GRADE Working Group approved in 2017 the formation of the GRADE Public Health Project Group. This article, which is the first output of this project group, is not GRADE guidance but a GRADE concept article. A GRADE concept article is a new type of communication from the GRADE Working Group that discusses the need for conceptual advances in GRADE methodology.

This article reports the results of activities undertaken to identify some of the challenges relating to GRADE and public health guidelines, specifically through an initial stakeholder workshop and a scoping review. We propose priority areas for further consideration and explain the rationale for these choices. We present four case studies of public health guidelines and systematic reviews (Case study 1, Case study 2, Case study 3, Case study 4) to illustrate how these challenges have been encountered in practice. The article concludes by summarizing the types of solutions that may be proposed for these challenges and the next steps for the GRADE Public Health Group.

Case study 1 **Cochrane systematic reviews on unconditional cash transfers**

**Table:** 

Contributed by: Stefan Lhachimi
Background
Unconditional cash transfers (UCTs, money provided without obligation) are an intervention that addresses a key social determinant of health, i.e., income. Two recent Cochrane reviews [54,55] assessed the health effects of UCTs: the first in the context of (humanitarian or natural) disasters and the second as a social protection intervention for reducing poverty and vulnerabilities (e.g., orphanhood, old age, or HIV infection). Both reviews included studies conducted in low- and middle-income countries only and had as primary outcomes the use of health services and health outcomes. Secondary outcomes were relevant social determinants of health (e.g., assets, education, labor force participation, parenting quality, and extreme poverty) and health care expenditure.
Examples of challenges
Challenges 2 and 3: Most studies included in the review of UCTs as a social protection intervention were conducted by economists. Outcome reporting in the economic literature may not take into account considerations necessary for evidence synthesis, such as choosing and defining outcomes in common with already existing studies on the same topic or reporting intracluster correlation coefficients in cluster randomized trials. Some studies have dozens of very similar outcomes (e.g., number of goats, number of cows, number of chickens, etc.), making it difficult to choose which one to report in a systematic review. Moreover, for many outcome measures used (in particular indices of food security and diet diversity), no agreed minimal important difference exists. The Summary of Findings table went through several iterations during the review process before agreement was reached on which and how many outcomes to report.
Challenges 4 and 5: In the review of UCTs for disaster relief, no high-quality evidence was identified. In addition, all included studies investigated UCTs as a response to only one type of disaster, namely droughts, which are different from most disasters in that droughts can be anticipated with a relatively long lead time before the consequences of the disaster materialize. Accordingly, the evidence was seriously indirect as well as low quality. Nevertheless, the UN at the World Humanitarian Summit in Istanbul 2016 called for making UCTs the default option for help during disasters. Intuitively, this seems plausible as UCTs in a short-term disaster context are (compared to in-kind transfers) swift and fairly easily to administer. However, this view does not account for potential crowding out effects of UCTs, i.e., unintended negative effects of UCT provision on other types of financial assistance during disasters.

Case study 2 **Cochrane systematic reviews to inform WHO malaria vector control guidelines**

**Table:** 

Contributed by: Leslie Choi
Background
In 2016, the WHO commissioned the development of new guidelines for malaria vector control [56] in partnership with the Cochrane Infectious Diseases Group (CIDG) based at the Liverpool School of Tropical Medicine. CIDG contributed eight systematic reviews [[57], [58], [59], [60], [61], [62], [63], [64]] (five de novo, two updates, and one previously published review) that were used to inform the recommendations made in these guidelines.
Examples of challenges
Challenges 2 and 3: Vector control tools are typically public health interventions distributed at a community level. To evaluate efficacy at a community level, appropriate study designs with applicable outcomes are required. The main challenge encountered with these guidelines is how to tailor the guidelines for the correct target audience. Are they for individuals wanting to protect themselves from malaria or for national malaria program planners? Paradoxically, increased protection for some individuals may translate into increased risk for others in the community who are not as well protected. Leading on from this, it is difficult to assess whether the study evidence included in the systematic reviews demonstrates community protection. For example, the systematic review on topical repellents combined studies that distributed the intervention at an individual level and those that distributed at a community level. Therefore, it was unclear if the conclusions drawn from that review were applicable to both contexts.
Challenge 4: Some modelling studies have suggested that poor coverage of vector control tools leads to more harm than good in a community by protecting a few individuals at the expense of the majority. However, these findings were difficult to capture and reconcile with the evidence from RCTs within the Summary of Findings and by extension the Evidence to Decision (EtD) framework.

Case study 3 **Czech national public health guideline on 1-day surgery**

**Table:** 

Contributed by: Miloslav Klugar
Background
One-day surgery was identified as a public health topic within the national Clinical Practice Guidelines project by the Guarantor Committee (representatives of important policymakers in the Czech Republic from the Ministry of Health, Institute of Health Information and Statistics, Czech Health Research Council, and health insurance organizations). One-day surgery has various definitions in different health systems worldwide; the definition for the Czech health system agreed by guideline panellists is “surgical performance (diagnostic and therapeutic) for hospitalization not exceeding 1 day (up to 24 hours of hospitalization, including one overnight stay).” In the Czech Republic, there are several surgical procedures in some hospitals in the 1-day surgery regime, while in other hospitals patients may be routinely hospitalized for the same surgical procedures for 3-6 days. One-day surgery clearly involves surgeons as a key audience, but also health care users, health care providers, and policymakers. In addition, following GRADE guidance, it was clear that social work and community care stakeholders needed to be engaged, as timely discharge from hospital and patient safety following discharge depend on social and community care.
Examples of challenges
Challenge 1: Initially the guideline panel membership included only surgeons and methodologists and the focus was on identifying which surgical procedures should be covered by 1-day surgery. There was initial resistance to inviting allied health professionals, patients, and other relevant stakeholders to join the panel. Furthermore, after framing the guideline questions the panel realized that social work/community care stakeholders should also be on the panel and that representation was needed from health and social insurance organizations, as well as homecare organizations. Identifying representatives from different organizations/sectors and managing the work of such a diverse guideline panel presented a challenge.
Challenge 2: Although the Population, Intervention, Comparison, Outcome format is straightforward in its application to public health questions, the panel faced a challenge in prioritizing clinical versus social and community care outcomes, particularly in terms of the identification and management of postsurgical complications and patient safety after discharge from hospital.

Case study 4 **European Breast Guidelines**

**Table:** 

Contributed by: Jesús López Alcalde and Zuleika Saz Parkinson
Background
The European Commission's Joint Research Center (JRC) supports European Union policies with independent scientific evidence [65]. The JRC coordinates the development of the European guidelines for breast cancer screening and diagnosis [66] (in short, the European Breast Guidelines). These guidelines follow GRADE [67]. The European Breast Guidelines provide recommendations on population-based breast cancer screening programs and diagnostic procedures for breast cancer [10,68,69]. The target audience of the guidelines is heterogeneous, covering health care providers, service users, and policymakers.
Examples of challenges
Challenges 1 and 2: To define the perspective of the European Breast Guidelines was not straightforward. Some questions are purely clinical, taking an individual perspective, while others have a population perspective. These aspects influence the selection of the guideline questions and outcomes. The prioritization of the outcomes to determine the effects of breast cancer screening programs is also challenging; some panellists may take the safety of screening interventions for granted and be reluctant to include harms as outcomes.
Challenges 3 and 4: The GRADE approach requires a multidisciplinary team with specific skills, which may not be available in all public health institutions. Therefore, certain tasks must be outsourced. The first guideline developed with GRADE in a public health institution can be logistically complicated, but the replication in future guidelines is expected to be more straightforward.
Challenge 5: As RCTs for some questions are scarce, or even thought to be unethical, the use of GRADE is expected to generate conditional recommendations. The misconception that GRADE gives too much weight to RCT evidence may partly explain this.

### 1.2 Background

Public health is concerned with “preventing disease, prolonging life, and promoting health through the organized efforts of society.” [4]. Public health encompasses three domains—health protection, health services, and health improvement [5]—within a disciplinary approach which recognizes the impact of social determinants on individual and population health outcomes and the importance of reducing health inequalities. Decisions made by public health practitioners, policymakers, and organizations may relate to interventions that are not implemented at the individual level, including health system reform, regulation of unhealthy commodities (such as taxation of alcohol or sugar-sweetened beverages), infrastructure development, and social security policies. Depending on the approach taken, policy goals may be competing or may produce contradictory results, for example, when addressing health inequalities [6]. Decisions about such interventions and situations involve stakeholders from many different professions and organizations. Consensus on critical outcomes may be challenging to achieve and evidence from well-conducted randomized studies may be lacking. However, despite any lack of randomized studies, decisions must be based on the best available evidence.

In this context, the GRADE approach has strengths which are well recognized. GRADE's structured and transparent methods for integrating evidence into decisions have been tested and implemented by guideline developers worldwide. Previous research has reported the adoption and further development of GRADE methods in public health contexts [[7], [8], [9], [10], [11]]. Previous research has also investigated stakeholder views and experiences with GRADE and confirmed that GRADE principles and processes are applicable to public health systematic reviews and guidelines [[12], [13], [14]]. However, challenges have also been identified for which various solutions have been proposed [12,[14], [15], [16], [17], [18], [19], [20]]. These challenges, along with the existence of multiple proposed solutions, represent areas in which further elaboration of GRADE guidance and production of exemplars may be beneficial to ensure consistent application of the methodology, support the production of high-quality systematic reviews and evidence-based guidelines, and allow for further improvement of the GRADE system.

To explore experiences of GRADE users and lay the foundations for a GRADE public health project group, guideline developers and systematic reviewers were invited to participate in a workshop on GRADE for public health at the Global Evidence Summit in Cape Town, South Africa, in September 2017. Twenty participants contributed to a facilitated discussion of challenges encountered when applying GRADE to public health topics. Participants reported issues with agreeing on critical outcomes, assessing certainty when the body of evidence includes findings from disciplines other than health, making appropriate use of nonrandomized studies, and addressing stakeholder concerns about weak or conditional recommendations. In addition, participants raised the issue of the broad range of stakeholders involved in public health decision-making and how to address the differences and competing interests that may arise when guidelines apply to sectors other than health. Further challenges in applying GRADE included the need to integrate the perspectives of different disciplines and professions in areas such as planning, housing, transport, and social security; the need to address competing policy priorities and resource considerations across governmental agencies and geographical boundaries; and the involvement of both public sector and commercial interests.

The formation of the GRADE Public Health project group was subsequently approved by the GRADE Guidance Group in October 2017 with the overarching goal to advance GRADE methodology in the development of public health guidelines. The work of the GRADE Public Health Group builds on previously developed and in-development GRADE guidance and related research on topics including equity, complex interventions, modeling studies, nonrandomized studies (NRS), and environmental and occupational health [[21], [22], [23], [24], [25], [26], [27]]. Here, we particularly focus on interventions that impact on whole populations or large population groups (i.e., are not delivered at the individual level or whose benefits are realized at a population level). Examples of such interventions include regulation of unhealthy commodities (e.g., restrictions on transfats in processed foods and minimum unit pricing of alcohol); the provision of new infrastructure (e.g., clean water and sanitation, affordable housing, and modifications to transport); vaccination programs; and health system reforms (e.g., the integration of health and social care services).

## 2 Methods

We conducted a scoping review with the aim of investigating (i) experiences of applying GRADE within public health systematic reviews and guideline development and (ii) existing research activity in this area. We included peer-reviewed published research on experiences, barriers, and facilitators encountered in public health contexts in the adoption of GRADE. We did not include methodological or technical documents that reported only the methods and not the experiences or reflections of reviewers or developers. We searched Medline and Embase (date range 2000–2018; see online supplement for search strategies); used the snowballing technique to identify additional relevant studies; searched the reference lists of included articles; and contacted experts in the field. We performed a narrative synthesis using thematic analysis to identify key issues and challenges reported in the literature. We then developed the analysis through an iterative process of consultation and discussion conducted with GRADE Working Group members electronically and at three GRADE Working Group meetings.

## 3 Results

We identified nine studies [[12], [13], [14], [15], [16],[28], [29], [30], [31]] that specifically described the experiences of guideline development agencies or systematic review groups in applying GRADE to public health topics.

Rehfuess and Akl gathered insights from 25 stakeholders in 12 organizations about their experiences and views of using GRADE in the field of public health [14]. They grouped the challenges identified by participants into six categories:

• Complexity of public health interventions.
• Choice of outcomes and outcome measures.
• Ability to discriminate between different types of observational studies.
• Use of nonepidemiological evidence.
• GRADE terminology.
• GRADE and guideline development process.

Issues relating to GRADE terminology included definitions of “quality of evidence,” the use of the term “observational study,” and different understandings of terms such as “low-quality evidence” and “weak recommendations.” Participants reported challenges relating to the methodological difficulties of systematically reviewing nonrandomized studies, applying GRADE criteria to narrative synthesis in the absence of a pooled effect estimate, difficulties with the evidence-to-recommendations process, and concerns about the resources required to implement GRADE methodology [14].

Five reports [12,13,15,28,30] have described experiences of using GRADE in the context of World Health Organization (WHO) guideline development. Akl et al reported their experiences of applying GRADE in the development of a WHO Department of HIV/AIDS guideline on public health interventions for men who have sex with men and transgender people [12]. They describe the solutions they developed within a single guideline for the following nine challenges:

• Heterogenous and complex interventions.
• Paucity of trial data.
• Selecting outcomes of interest.
• Using indirect evidence.
• Integrating values and preferences.
• Considering resource use.
• Addressing social and legal barriers.
• Wording of recommendations.
• Developing global guidelines.

Dedios et al. evaluated the methodological quality and implementability of guidelines produced by the WHO Department of Nutrition for Health and Development [15]. They suggested that difficulties encountered in their evaluation may reflect limitations of GRADE guidance, specifically citing NRS, time requirements, and wording or strength of recommendations as issues. Alexander et al. investigated why WHO guideline developers make “discordant recommendations,” i.e., strong recommendations based on low or very low certainty evidence. Their interviews with guideline panel members identified several challenges or barriers to GRADE application, including insufficient guidance on prioritizing outcomes and rating NRS, different professional backgrounds among panel members, and a political environment that favors strong recommendations [13]. In a further study by Alexander et al, interviews with GRADE methodologists also identified the political environment as a driver of “discordant” recommendations as well as issues with the management of conflicts of interest, urgent timescales, and the feasibility of implementing recommendations [28]. Most recently, Gopinathan and Hoffman interviewed 35 senior WHO staff on their views about changes in WHO guideline development because its processes were reformed starting in 2007 [30]. Among several themes identified in the interviews, challenges were identified relating to the assessment of NRS and qualitative studies, concerns which the authors note were subsequently addressed with the introduction of ROBINS-I [32] and GRADE-CERQual [33].

Two reports [16,31] relate to guidelines or projects at the European Center for Disease Prevention and Control (ECDC). Within a review of evidence-based guideline methodology, the authors described issues with GRADE's terminology and treatment of NRS and noted an absence of guidance relating to economic and epidemiological models, studies of incidence and prevalence of disease, and microbiological studies. They further noted the need to integrate other aspects of decision-making with GRADE processes, such as prioritization of topics and selection of experts [16]. The ECDC Project on a Framework for Rating Evidence in Public Health subsequently reported the discussions and conclusions of an international expert meeting on evaluating and grading evidence on infectious disease prevention and control [31]. This report concluded that GRADE can be applied successfully to guideline questions concerning the incidence and prevalence of diseases and suggested that some types of nonrandomized evidence, such as a body of evidence from interrupted time series (ITS) designs, should be rated more highly in GRADE assessments. This is an area for which the GRADE Working Group has sought examples and operationalization since its inception but failed to receive or identify clear methodological suggestions that would allow rating ITS higher.

Finally, Burford et al. (2012) summarized the benefits and challenges when applying GRADE to Cochrane public health systematic reviews [29]. These challenges related to NRS and rating certainty of evidence, as well as what to do in the absence of a pooled effect size. The article addressed some misconceptions relating to initial ratings of randomized and observational studies and noted that concerns about low certainty of evidence are not unique to public health. The authors further noted that GRADE is particularly helpful in addressing certain issues commonly encountered when synthesizing public health evidence, including the importance of context and the heterogeneity that arises when combining findings from different public health programs and health systems.

The studies identified in the scoping review range in publication date from 2011 to 2018. During this period, developments in GRADE methodology occurred that addressed many, if not most, of the challenges identified. For example, GRADE changed its terminology about the quality of evidence to be more flexible. Furthermore, GRADE had already altered the traditional evidence hierarchy by allowing a body of evidence from observational studies to be rated as high-quality evidence. In addition, articles have since addressed the use of GRADE in the absence of meta-analyses [18,34]. Overall, the existing literature is consistent in identifying certain challenges in using GRADE within public health systematic reviews and guidelines, particularly regarding diverse stakeholder perspectives within political environments, rating the certainty of nonrandomized evidence and wording or strength of recommendations. Most of the included studies make suggestions or recommendations to address these issues, and many solutions have been developed. However, additional solutions require further investigation, development, and testing to ensure a systematic approach that is coherent with existing GRADE guidance while also meeting the needs of stakeholders.

## 4 Analysis: key challenges and solutions when applying GRADE guidance to public health topics

**Fig. 1:**
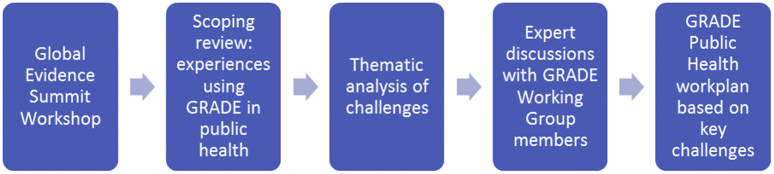
Process of developing the scope and priorities of the GRADE Public Health Group.

To formulate an approach to addressing the issues identified through the scoping review, the GRADE Public Health Group organized the challenges into themes, mapped these to current GRADE Working Group activities, identified gaps, discussed and presented potential activities and outputs, and reached consensus on a program of work including publications, contributions to other project groups, and training. The project group also identified and discussed case studies through which the relevance and applicability of the five themes were explored and confirmed in a variety of public health contexts, including both systematic reviews and guidelines. Figure 1 summarizes the process of developing the scope and priorities of the GRADE Public Health Group, leading to a focus on five key challenges.

The five key challenges are described narratively below. Table 1 summarizes the analysis of the challenges, gaps, and the project group response, including existing solutions, planned outputs, and other activities. Case study 1, Case study 2, Case study 3, Case study 4 present the case studies, which have been selected to represent a diversity of public health topics (social determinants of health, infectious diseases, organization of health care, and screening) drawn from both systematic reviews and guidelines (ten Cochrane reviews, one national public health guideline, and one pan-national guideline development program).

**Table 1:** Key challenges, examples, and proposed solutions in applying GRADE guidance to public health topics

Challenge	Examples	Solutions
1. Incorporating diverse perspectives	Convening guideline panels with diverse perspectives from different sectors, e.g., Czech national public health guideline on 1-day surgery (Box 3) Combining individual and population perspectives, e.g., malaria vector control reviews (Box 2) and European Breast Guidelines (Box 4)	Forthcoming GRADE Public Health article, Addressing social determinants in public health systematic reviews and guidelines using GRADE Adapt GRADE training materials for nonhealth and policy audiences
2. Selecting and prioritizing outcomes	Addressing individual and population outcomes in the same guideline or review, particularly when benefits and harms differ depending on the perspective taken, e.g., Cochrane reviews and WHO guideline on malaria vector control (Box 2) Achieving consensus on critical outcomes across different sectors, e.g., health care, social work, community care, and insurers in Czech national public health guideline on 1-day surgery (Box 3) Health vs. nonhealth outcomes, e.g., in transport policy	
3. Interpreting outcomes and identifying a threshold for decision-making	Outcomes that have no defined thresholds, e.g., Cochrane reviews of unconditional cash transfers (Box 1) Small changes may have important health impacts at population level but not at individual level, e.g., taxation to address health externalities Different thresholds will have implications for modelling of the population health effects of policies and public health interventions	Forthcoming GRADE Public Health article, Considerations when applying GRADE to population-level outcomes in public health systematic reviews and guidelines
4. Assessing certainty of evidence from diverse sources, including nonrandomized studies	Cochrane reviews of environmental interventions to reduce sugar-sweetened beverage consumption and interventions to reduce ambient air pollution found large differences in internal validity both within and across NRS Natural experiments may involve randomization or allocation that is “as good as” random, and may be the best possible evidence for a policy-related question but are treated as nonrandomized or observational studies and generally start with a rating of low-quality evidence	Contribute worked examples from public health reviews and guidelines to other GRADE project groups, especially the GRADE NRS group Operationalize criteria for when interrupted time series may avoid typical selection and confounding bias and be rated as moderate certainty
5. Addressing implications for decision makers, including concerns about conditional recommendations	Making strong recommendations despite very low certainty evidence, e.g., unconditional cash transfers for disaster relief (Box 1)	Collaboration with GRADE EtD group ongoing since May 2019

### 4.1 Challenge 1: incorporating diverse perspectives

Many health guidelines are intended for multidisciplinary audiences, including different health professions, health care managers, health policymakers, patients, and carers. The Health in All Policies approach is arguably where this becomes most challenging, for example, when considering the health impacts of migration policy [35]. In addition, to address the wider determinants of health, public health guidelines may be targeted at professionals, policymakers, and other stakeholders from nonhealth disciplines and sectors whose perspectives on health evidence may vary; indeed, sometimes the primary responsibility for financing and/or implementing public health interventions lies within these sectors. For example, the National Institute for Health and Care Excellence guideline on physical activity and the environment is aimed at local government and their contractors, employers, and community organizations responsible for public spaces, housing authorities, public transport planners and providers, and organizations that support people with limited mobility [36]. The way that such varied audiences frame policy questions, the extent to which these audiences value health protection and improvement, and the priorities placed on various policy approaches may all differ substantially [37], but need to be understood if the guideline is to achieve its aims. Furthermore, different professions and stakeholder groups may represent different “cultures of evidence” in which legislation, regulations, and other contextual factors may take priority over scientific research [38].

### 4.2 Challenge 2: selecting and prioritizing outcomes

Differences in perspectives and in cultures of evidence also translate into challenges in selecting relevant outcomes and agreeing which outcomes are critical to decision-making. For example, in transportation policy any recommendations about traffic management would ideally consider all relevant outcomes, including respiratory health, changes in physical activity levels, emergency admissions, and safety for vulnerable road users. However, transport policymakers might not view these outcomes as critical when weighed against traffic flow, commuting times, road traffic collisions, costs, provision of goods, or public opinion. Relatedly, challenges may arise when benefits are realized to a greater extent by communities than by individuals who receive treatment (e.g., immunizations which lead to herd immunity) [26]. A further challenge relates to the potential trade-off between population health and health equity, in which a population health benefit may be achieved at the expense of widening inequalities. For example, a public health intervention such as screening or wellness checks may succeed in improving a population's overall morbidity and mortality, but if the improved outcomes are predominantly achieved in a subgroup of higher socioeconomic status (SES), health inequality may inadvertently be increased by widening the difference between the outcomes of high versus low SES subgroups. The GRADE equity guidelines [21,24,26,27] are helpful in addressing this challenge. Finally, advancing health in the context of the Sustainable Development Goals implies that health benefits may need to be weighed against broader environmental, economic, and social impacts and their potential interactions [[39], [40], [41]].

### 4.3 Challenge 3: interpreting outcomes and identifying a threshold for decision-making

The interpretation of evidence for a given outcome needs to be informed by knowledge of what change in that outcome would make a meaningful difference in a given context. A change in a health outcome that might be deemed too small to be of importance to a patient may be perceived differently from a whole population perspective, where small effects in many individuals may be magnified to differences that are important for public health interventions and policy [42], as can be seen, for example, in population-wide salt reduction programs [43]. Furthermore, many interventions at the population level are implemented for primarily nonhealth reasons but act on key determinants of health, for example, transport infrastructure, housing, and general taxation, and therefore may have profound health and health equity impacts. In these circumstances, the effect size of an intervention may not be as informative for decision-making as the expected direction of overall health impact (e.g., likely results in health gains or health harms). Relatedly, equipoise may not exist for the primary nonhealth outcome [44]. These considerations of population-level impact, social determinants of health and inequalities, and lack of equipoise may all influence views on what constitutes an important difference and the required degree of precision to support decision-making.

### 4.4 Challenge 4: assessing certainty of evidence from diverse sources, including NRS

In public health, where randomized studies are less common and often infeasible in comparison with other areas of health, some types of NRS may provide greater certainty than others when investigating the health effects of policy or social interventions. For example, natural experiment studies may address selection bias and confounding through designs such as ITS or regression discontinuity, which may support stronger causal inference than other observational designs such as cohort and case-control studies [45]. Two recent Cochrane reviews, one on environmental interventions to reduce sugar-sweetened beverage consumption [46] and one on interventions to reduce ambient air pollution [47] used nonrandomized intervention studies where no randomized studies were available; however, there were large differences in internal validity both within and across NRS. Existing GRADE guidance has noted that the potential exists for different NRS designs to provide moderate-quality evidence but that examples are yet to be identified [25]. An active research agenda exists in the area of NRS and evidence synthesis, including the activity of the GRADE NRS group, the development of ROBINS-I within the Cochrane Risk of Bias Methods Group, and further adaptations of ROBINS-I for different NRS designs [32,48]. Addressing this challenge by identifying relevant examples from public health policy and applying these new tools therefore seems a promising catalyst through which solutions to other challenges may also emerge.

### 4.5 Challenge 5: addressing implications for decision makers, including concerns about conditional recommendations

As demonstrated in the scoping review, the predominance of “weak” recommendations in public health guidelines has been of concern to guideline developers and policymakers. GRADE has addressed this issue by changing the terminology (weak recommendations are in public health primarily labeled conditional and the conditions should be specified). It is already recognized that public health produces several situations in which action may be strongly recommended despite low or very low certainty, including:

• Life-threatening situations.
• Uncertain benefit but certain harm.
• Potential equivalence of effectiveness in which one option is clearly more or less risky or costly.
• Potential for catastrophic harm [49].

Early strong recommendations in the context of very low certainty evidence emphasized that GRADE had recognized this problem [50]. However, examples may still be encountered, such as in fiscal policy, where economists might strongly recommend austerity policies while public health might produce a weak recommendation in terms of health outcomes. Grading recommendations as “conditional” rather than “weak” may partially address this issue, but guideline developers may also need to better contextualize and ground such recommendations in “what else we might know.” A Bayesian decision theory approach has been proposed in the context of this problem [[51], [52], [53]] and could be further explored. A key challenge for GRADE in public health is therefore to identify how to reconcile the tension between the methodologically correct presentation of evidence and recommendations as per GRADE and the implications of strong vs. conditional recommendations from the perspective of decision makers in political environments.

[Case study 1, Case study 2, Case study 3, Case study 4: Case studies: four examples of challenges of applying GRADE in public health systematic reviews and guidelines].

## 5 Discussion and conclusion

The views sourced from a participatory workshop, a scoping review, and four case studies suggest that key public health-related challenges in application of GRADE methodology include the incorporation of stakeholder perspectives from outside the health sector; agreement on the interpretation and prioritization of diverse outcomes; definition and interpretation of thresholds for population health and nonhealth outcomes; assessing the certainty of evidence from diverse sources and NRS designs; and addressing implications for decision makers, including the presentation of strength of recommendations to ensure utility and acceptability for stakeholders.

These challenges may be categorized into three different types, as per the different types of solutions that may be proposed. The first type of solution is to identify the training needs of public health guideline developers (and their stakeholders) in understanding and using the GRADE concept, i.e., as per existing guidance. As different disciplines or “cultures of evidence” come together in public health, it will take time to speak a common (GRADE) language. The second type of solution has to do with applying existing GRADE guidance, including current developments, to the public health context. As identified within the scoping review, most of the existing GRADE guidance, both on rating certainty of the evidence and moving from evidence to decision, is already applicable to the public health context but would benefit from translation and examples, so that its relevance to public health practice and policy can be more readily recognized and a population perspective can be consistently reflected in all steps. The third type of solution is to advance the GRADE methodology where existing guidance falls short, i.e., to develop new GRADE guidance where necessary and appropriate.

The GRADE Public Health Group has considered the challenges in the context of other GRADE outputs and project groups, as well as other ongoing research in guideline methodology [41,70], with the aim of avoiding unnecessary duplication of effort. The solutions proposed by the group include the following package of work:

• Further GRADE concept articles or guidance to address social determinants in public health systematic reviews and guidelines using GRADE, considerations when applying GRADE to population-level outcomes and criteria to assess certainty of interrupted time series.
• The ongoing development and dissemination of detailed examples of the application of GRADE to public health topics in coordination with other GRADE project groups, including the NRS and EtD groups.
• Adaptation of GRADE training materials for public health and public policy audiences.

By providing a single, carefully documented, rigorous, and transparent method of evaluating evidence, GRADE serves to increase the trustworthiness, implementation, and adaptability of systematic reviews and guidelines, while also helping to reduce research waste. It is therefore our aim to address and mitigate any barriers to using GRADE in public health and, through the work of the GRADE Public Health group, to build bridges and increase mutual understanding across the wide range of policy and research areas that intersect in the population health context.

## CRediT authorship contribution statement

**Michele Hilton Boon:** Conceptualization, Methodology, Writing - original draft, Writing - review & editing. **Hilary Thomson:** Conceptualization, Writing - review & editing. **Beth Shaw:** Conceptualization, Investigation, Writing - review & editing. **Elie A. Akl:** Conceptualization, Methodology, Writing - review & editing. **Stefan K. Lhachimi:** Writing - original draft, Writing - review & editing. **Jesús López-Alcalde:** Writing - original draft, Writing - review & editing. **Miloslav Klugar:** Writing - original draft, Writing - review & editing. **Leslie Choi:** Writing - original draft, Writing - review & editing. **Zuleika Saz-Parkinson:** Writing - original draft, Writing - review & editing. **Reem A. Mustafa:** Methodology, Writing - review & editing. **Miranda W. Langendam:** Methodology, Writing - review & editing. **Olivia Crane:** Writing - review & editing. **Rebecca L. Morgan:** Writing - review & editing. **Eva Rehfuess:** Writing - review & editing. **Bradley C. Johnston:** Writing - review & editing. **Lee Yee Chong:** Writing - review & editing. **Gordon H. Guyatt:** Methodology, Writing - review & editing. **Holger J. Schünemann:** Conceptualization, Methodology, Writing - review & editing. **Srinivasa Vittal Katikireddi:** Conceptualization, Methodology, Investigation, Writing - review & editing.

## Supplementary data

Data Profile
