# Supplementary material for: Challenges in applying the GRADE approach in public health guidelines and systematic reviews: a concept article from the GRADE Public Health Group
Source: J Clin Epidemiol. 2021 Jul;135:42–53. doi: 10.1016/j.jclinepi.2021.01.001 (PMC8352629; doi:10.1016/j.jclinepi.2021.01.001)
Supplement: Appendix [file mmc2.docx]

Online Supplement

Scoping search strategy for “Challenges in applying the GRADE approach in public health guidelines and systematic reviews: A concept paper from the GRADE Public Health Group”

Medline strategy

1. Practice Guidelines as Topic/

2. GRADE.ti,ab.

3. public health/ or epidemiology/ or preventive medicine/ or environmental medicine/ or occupational medicine/ or social medicine/ or tropical medicine/ or vaccinology/

4. Health Policy/ or Health Status Disparities/ or "Social Determinants of Health"/ or Socioeconomic Factors/

5. 1 and 2

6. 3 or 4

7. 5 and 6

Embase strategy

1. Practice Guidelines as Topic/

2. GRADE.ti,ab.

3. *practice guideline/

4. (1 or 3) and 2

5. *public health/ or *social medicine/

6. "social determinants of health"/

7. health care policy/

8. or/5-7

9. 4 and 8
